# Electrophysiological Proxy of Cognitive Reserve Index

**DOI:** 10.3389/fnhum.2021.690856

**Published:** 2021-07-08

**Authors:** Elvira Khachatryan, Benjamin Wittevrongel, Matej Perovnik, Jos Tournoy, Birgitte Schoenmakers, Marc M. Van Hulle

**Affiliations:** ^1^Laboratory for Neuro- and Psychophysiology, Department of Neurosciences, KU Leuven, Leuven, Belgium; ^2^Leuven Brain Institute, Leuven, Belgium; ^3^Faculty of Medicine, University of Ljubljana, Ljubljana, Slovenia; ^4^Department of Neurology, University Medical Center, Ljubljana, Slovenia; ^5^Department of Geriatrics and Gerontology, KU Leuven, Leuven, Belgium; ^6^Academic Centre of General Practice, KU Leuven, Leuven, Belgium

**Keywords:** EEG, cognitive reserve, aging, P300 - event related potential, oddball paradigm

## Abstract

Cognitive reserve (CR) postulates that individual differences in task performance can be attributed to differences in the brain’s ability to recruit additional networks or adopt alternative cognitive strategies. Variables that are descriptive of lifetime experience such as socioeconomic status, educational attainment, and leisure activity are common proxies of CR. CR is mostly studied using neuroimaging techniques such as functional MRI (fMRI) in which case individuals with a higher CR were observed to activate a smaller brain network compared to individuals with a lower CR, when performing a task equally effectively (higher efficiency), and electroencephalography (EEG) where a particular EEG component (P300) that reflects the attention and working memory load, has been targeted. Despite the contribution of multiple factors such as age, education (formal and informal), working, leisure, and household activities in CR formation, most neuroimaging studies, and those using EEG in particular, focus on formal education level only. The aim of the current EEG study is to investigate how the P300 component, evoked in response to an oddball paradigm, is associated with other components of CR besides education, such as working and leisure activity in older adults. We have used hereto a recently introduced CR index questionnaire (CRIq) that quantifies both professional and leisure activities in terms of their cognitive demand and number of years practiced, as well as a data-driven approach for EEG analysis. We observed complex relationships between CRIq subcomponents and P300 characteristics. These results are especially important given that, unlike previous studies, our measurements (P300 and CRIq) do not require active use of the same executive function and, thus, render our results free of a collinearity bias.

## Introduction

Cognitive reserve (CR) is a concept that was introduced to explain the discrepancy between an individual’s actual cognitive performance, measured by cognitive tests, and the expected one based on structural brain changes observed on neuroimaging scans (Stern, 2009). It was initially suggested that CR acts as a compensatory mechanism enabling the brain to recruit additional networks to improve task performance. Later, it was shown that CR not only compensates for certain degrees of structural brain changes but also aids in the development of alternative strategies to more efficiently (e.g., faster and more accurately) complete a cognitive task. Physiologically, individuals with higher CR, among others, are demonstrated to have more synaptic connections between neurons compared to low-CR individuals (Stern, 2002; Esiri and Chance, 2012). CR accumulates during the course of a lifetime as a result of formal and informal experiences, including but not limited to educational attainment, type of employment, number of languages mastered, and hobbies. Importantly, each individual, whether old or young, has a certain level of CR. However, when brain damage exceeds a certain threshold, the compensatory mechanism provided by CR fails and the clinical manifestation of brain damage comes into the picture (Stern et al., 1995; Van Loenhoud et al., 2019).

In older individuals, CR is often evaluated by combining the outcomes of structural brain imaging and cognitive tests (Mungas et al., 2010; Reed et al., 2010; Zahodne et al., 2013). Under the presence of brain changes (i.e., atrophy), a good score on the cognitive tests is an indication of a compensatory CR effect. On the other hand, when brain changes are absent and cognitive test results exhibit a ceiling effect, as for instance in healthy young individuals, the CR level is difficult to estimate. For these cases, functional neuroimaging techniques, such as functional MRI (fMRI), are being adopted to unveil the brain networks that are involved in the execution of a task (Anthony and Lin, 2017). The degree of neural activation in combination with the task performance level provides evidence of increased efficiency or compensation due to CR (Bozzali et al., 2015; Anthony and Lin, 2017; Conti et al., 2021). For instance, good task performance with a relatively low level of neural recruitment or spatially limited brain activations suggests high brain efficiency and therefore a high CR level, as is often observed in healthy young individuals (Conti et al., 2021). On the other hand, good task performance in combination with a high level of activation or the inclusion of additional brain networks indicates a compensatory mechanism related to the available CR, which is often seen in older individuals even in the presence of a (mild) brain atrophy (Colangeli et al., 2016; Anthony and Lin, 2017; Cabeza et al., 2018).

A recently introduced approach to evaluate CR is the so-called CR index questionnaire (CRIq), which takes into account both formal (education, work) and informal (hobby) activities (Nucci et al., 2012). Unlike other methods, the questionnaire is freely available, is easy to use in different settings, and manages to quantify the obtained results. However, the downside is that it is rather generic and empirical and does not reflect the underlying neurophysiological processes. It was introduced in several languages (Kartschmit et al., 2019) and was implemented in both young and older healthy adults (Nucci et al., 2012), as well as patients with cognitive decline (Garba et al., 2020). Given that there is no “gold standard” for CR assessment, the authors of CRIq were unable to validate it. They did, however, check the correlation between the CRI score and the participants’ intelligence level and observed a rather moderate correlation (0.45). Note that the latter is often used as a CR proxy (Caffò et al., 2016; MacPherson et al., 2017), despite being only partially representative for CR. While several studies suggest that the CRIq has a good test-retest reliability (Nucci et al., 2012; Kartschmit et al., 2019; Garba et al., 2020). Garba et al. (2020) questioned its validity when not observing a correlation between CRIq score and score on Wechsler Test of Adult Reading (WTAR) in patients with Alzheimer Disease (AD). However, it is important to note that in AD patients, as mentioned before, the reserve capacity is exhausted (Van Loenhoud et al., 2019) and, therefore, it would not be expected that the results obtained from CRIq would correlate with a task performance.

In addition to fMRI, electroencephalography (EEG) has been shown useful for the evaluation of CR. It can be considered complementary to the spatially-accurate fMRI as it measures cumulative post-synaptic activity in large neuronal populations with millisecond precision (Buzsáki et al., 2012). The most studied EEG component in the context of CR is the P300 event-related potential, a positive EEG deflection peaking around 300 ms in response to presenting a stimulus-of-interest (Soltani and Knight, 2000; Polich, 2012; Šneidere et al., 2020). It has been associated with both the individual’s mental capacity such as working memory (Saliasi et al., 2013; Juan et al., 2019) or verbal fluency (Francisco et al., 2019) and their socio-demographic background such as formal education level (Begum et al., 2014; Hasan et al., 2016). For instance, Amin et al. (2015) showed a positive correlation between the amplitude of the P300 component and memory recall, which they suggest to be indicative for CR. Francisco et al. (2019) observed such correlation between verbal fluency and P300 amplitude. Furthermore, a number of studies suggest a dependency of the P300 characteristics on the individual’s educational level, with most of them observing a lower P300 amplitude and larger latency for subjects with lower education levels compared to ones with higher levels (Begum et al., 2014; Hasan et al., 2016). Even though these results are promising in terms of promoting EEG for the evaluation of an individual’s CR-level, the mentioned studies merely focus on one contributor to the development of CR, being either education level (Begum et al., 2014; Hasan et al., 2016), verbal fluency (Francisco et al., 2019) or IQ level (Amin et al., 2015), among others, and fail to account for the potential complex contribution of multiple factors to the overall CR. For example, in the case of assessing CR only based on education level, an individual with a poor socioeconomic status who was unable to obtain university-level education, but who has built up a considerable CR due to a passion for reading, will still be considered to have a low CR-level. Only a few studies compared EEG-correlates of CR with a composite CR score (Šneidere et al., 2020). From these studies, Amodio et al. (2017), for instance, did not observe any relation between resting-state EEG and CRIq score in patients with hepatic encephalopathy, while Speer and Soldan (2015) showed an association between the P300 component in response to a working memory task and a CR score based on reading test results, verbal intelligence and years of education in both young and older participants. Here, the authors observed a smaller change in P300 amplitude and latency in individuals with a higher CR for increasing task difficulty compared to the ones with a lower CR. Importantly, this study recorded a P300 component in response to a working memory task. Since the latter is believed to be highly associated with verbal intelligence (Cantor et al., 1991), it is not clear whether the association between the EEG response and CR-score was direct or mediated *via* working memory. It has been repeatedly shown that P300 can reflect the individual’s working memory (Amin et al., 2015; Juan et al., 2019) and that its amplitude and latency changes with the working memory load (Saliasi et al., 2013; Juan et al., 2019; Miranda et al., 2020). Even though CR has a direct effect on working memory, as shown in both healthy individuals and patients with subjective cognitive decline and mild cognitive impairment (MCI; Mitchell et al., 2012; Lojo-Seoane et al., 2014, 2018), working memory is not the only cognitive activity strongly influenced by CR (Giogkaraki et al., 2013; Lojo-Seoane et al., 2014). However, when considering a working memory task to evoke the P300 component, the outcome could be confounded by collinearity. Attention, on the other hand, even though closely related to working memory (Oberauer, 2019), does not exert a direct influence on the flexible processes of working memory. In this vein, we propose an attention task to evoke the P300 to ensure that the observed relationships between the latter and CR are not mediated *via* working memory but are rather genuine.

In the current study, we compare the characteristics of the P300 component in response to an attention task with the CRI-subscores obtained from the CRIq in older adults. This will provide us with an opportunity to consider the effect of hobby’s and outwork activities (e.g., volunteering) in the formation of CR, as well as to avoid a large influence of working memory on the recorded P300 component.

## Methods and Materials

### Participants

We recruited 20 Flemish-speaking non-demented older adults (ranging between 52 and 89 years old, all right-handed) from the community. Any current or previous major neurological or psychiatric event (e.g., stroke, parkinsonism, brain tumors, major depression, or hallucinations) was considered as exclusion criteria. Additional exclusion criteria were impaired vision or hearing that would preclude the subject from following the instructions. Inclusion criteria for recruitment were age above 50 years old and absence of dementia assessed based on independence in daily life by a neurologist (EK) and corroborated by a close relative. All subjects had a normal or corrected-to-normal vision. The study was approved by the Ethics Committee of the Leuven University Hospitals. All participants read and signed an informed consent form after being informed about the purpose and setting of the study, as well as how their data was going to be processed and stored (GDPR).

### Experimental Paradigm

All participants were screened using the Dutch version of the standardized Mini-Mental State Examination (MMSE; Kok and Verhey, 2002) and Clinical Dementia Rating (CDR) tests (Morris, 1997). We additionally administered the Montreal Cognitive Assessment (MoCA) test, as it has shown to be more sensitive for changes in cognition and detection of individuals with MCI (Nasreddine et al., 2005). We estimated the CR of individual participants using the cognitive reserve index questionnaire (CRIq; Nucci et al., 2012), which provides sub-scores for education, working attainment, and leisure activities, as well as a combined total score. For the education-sub-score, both formal education and vocational trainings that lasted at least 6 months were counted, while the working attainment sub-score depended on the level of mental demand and responsibilities the employment required, and the leisure activity sub-score included activities that would be performed weekly (e.g., chores, gardening, hobbies), monthly (e.g., volunteering, social events, and gatherings) and yearly (e.g., travel, reading books, and participation in conferences). Since we could assess these activities quantitatively, we were able to achieve our goal of assessing the influence of subjects’ informal activities on measured P300 characteristics.

**Figure 1:**
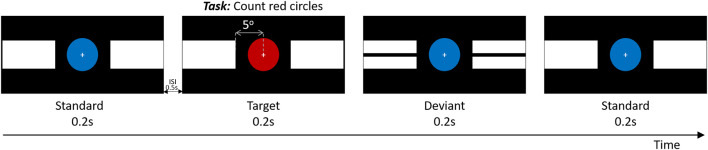
Schematic rendition of experimental paradigm. Timing is in seconds. ISI = interstimulus interval.

In addition to the neuropsychological tests described above, all participants performed a computer task with a simultaneous EEG recording during which they were sitting in a comfortable chair at a distance of approximately 70 cm from a 24-inch LCD monitor (ViewPixx EEG Canada, resolution 1,920 × 1,080) with a true refresh rate of 120 Hz. The stimulation paradigm consisted of three types of stimuli, namely a target, deviant, and standard stimulus (Figure 1), displayed in pseudorandom order for 200 ms with a jittered (±100 ms) inter-stimulus interval of 500 ms. The target stimulus consisted of a central red circle with two solid white horizontal blocks shown peripherally, on both sides. For the standard stimulus, the red circle was replaced by a blue circle, and the deviant stimulus consisted of a central blue circle and black line splitting the previously mentioned white blocks horizontally. In all three cases, the visual angle between the center of the attended central circle and the closest edge of the white horizontal blocks which are unattended (Figure 1) was 5^o^. The central circle spans a visual angle of 3.2^o^. The duty cycle of the target, deviant and standard stimuli was 1:1:3. Subjects were instructed to mentally count the occurrence of the targets (red circles). All subjects completed four rounds, in each of which between 20 and 25 target stimuli were presented. The total number of target stimuli across the four rounds was always 100. The stimulation was presented using the Matlab-based (version R2018a.) Psychophysics toolbox (Brainard, 1997) for precise timing. The entire experiment, including screening, EEG set-up, and breaks, lasted not longer than 1 h.

### EEG Acquisition

EEG data were acquired continuously using 62 active Ag/AgCl electrodes evenly distributed across the scalp at locations following the international 10/20 system. The ground and reference electrodes were placed at AFz and FCz, respectively. In order to ensure optimal contact between the subject’s skin and the electrodes, a drop of conductive gel was applied to each electrode. The impedance was kept below 5 kOhm throughout the recording. The EEG data were acquired at a sampling rate of 1 kHz using a Synamps RT device (Compumedics, Australia) and stored on a laptop for further analysis.

### EEG Data Processing

The obtained EEG data was offline re-referenced to the average of the mastoid electrodes (TP9 and TP10) and filtered between 0.2 and 15 Hz using a 4th order zero-phase Butterworth filter. Afterward, the EEG recording was cut into epochs starting from 100 ms before until 1,000 ms after the onset of each stimulus. Only epochs in response to the target and standard stimuli were used in the current study. The responses to deviant stimuli will be analyzed and processed in the scope of another study. The epochs were baselined to the average of the 100 ms pre-onset signal. The epochs for which the maximal amplitude exceeded ± 75 μV were rejected and the electrodes that had less than 50 remaining epochs per condition were also rejected. On average 1.05 (standard deviation = 1.23) channels were rejected per subject, ranging between 0 and 4 channels.

For the first step in the analysis, we performed a cluster-based permutation test (Maris and Oostenveld, 2007) using the Matlab-based *Fieldtrip* toolbox (Oostenveld et al., 2011) to find spatiotemporal clusters that exhibit a significant difference in response to the target and standard stimuli. We used a Monte-Carlo method to perform a significance probability mapping using the two-tailed independent *t-test* and max-sum as cluster statistics. At least two neighboring electrodes had to pass the significance threshold of 0.05 for it to be considered a cluster. The cluster-based procedure was repeated 2,000 times. The Supplementary Figure 1 shows the largest positive cluster and its spatio-temporal distribution for an individual subject. After the permutation test, the electrodes that were part of the largest positive cluster were isolated and an average ERP per condition was obtained by averaging the epochs in response to the target (or standard) stimulus, first across electrodes then across trials for each individual subject. The P300 effect was calculated by subtracting the per-subject average of standard epochs from the average of target epochs. Afterward, the peak amplitude and peak latency of the P300 effect were estimated as the maximal positive value and its corresponding latency between 250 and 850 ms post-onset. Note that this data-driven approach allows us to isolate the P300 component and to account for the spatial variability across subjects, which is especially important when investigating older participants as the P300 exhibits a frontal shift with age (van Dinteren et al., 2014).

### Statistical Analysis

A stepwise linear regression (Matlab function *stepwiselm*) model with only first level interactions was fitted with the aim to model the relationship between the questionnaire’s sub-scores (i.e., CRIq-subcomponents and MoCA) and the peak amplitude and latency of the P300-effect. As predictors, the following subject parameters and their first-level interactions were chosen: the sub-component scores for CRI-working, CRI-leisure, CRI-education, and the MoCA score. Since the total score of CRIq (CRI-total) is composed of its subcomponents and, our aim was to investigate the effect of each subcomponent on P300 characteristics, we did not include the total score in the model. The stepwiselm function uses forward and backward stepwise regression to determine the final model. We started with a model containing all predictors and iteratively updated the model by removing and/or adding individual predictors based on the F-statistics criterion (statistical significance). For removing the predictors, we used a default *p-value* of 0.1 and for adding them—a default *p-value* of 0.05. Note that the regression function operates in a way such that it does not observe all predictor combinations, thereby reducing the probability of overfitting. The significance level of the final regression model was set to 0.05.

## Results

All participants achieved scores that corresponded to the non-demented age-matched values of both MMSE and CDR tests: MMSE ≥27 (with the exclusion of one 89-year-old participant who scored 24 on MMSE) and CDR ≤0.5. Table 1 presents the results for demographics characteristics, MMSE, CDR, MoCA, and CRI-subcomponents in the studied group.

**Table 1:** Demographic and cognitive characteristics of the studied group.

	Mean (standard deviation)	Range
Age	68.6 (9.7)	52–89
Male/female	13/7	
Years of education	15.7 (3)	12–24
MMSE score	28.6 (1.6)	24–30
CDR	0.15 (0.2)	0–0.5
MoCA	26.6 (3.36)	18–30
CRI-total	129.6 (17.4)	93–167
CRI-education (CRI-e)	130.6 (20.6)	98–178
CRI-work (CRI-w)	120.3 (18.2)	88–161
CRI-leisure (CRI_l)	116.2 (16.6)	92–142

The cluster-based permutation test reveals on average 2.7 (ranging between 1 and 5) positive clusters per subject (i.e., clusters in which the amplitude in response to the target stimulus was larger than in response to the standard stimulus) within the first second after stimulus onset. The average peak amplitude and peak latency of the P300-effect is 7.8 μV (standard deviation (std) is 3.2 μV) and 503.15 ms (std is 153 ms), respectively. Supplementary Figure 2 illustrates the P300 effect for each electrode from subject S1 and Supplementary Figure 3 shows the average ERP-trace for electrodes Pz, Cz, and Fz.

**Figure 2:**
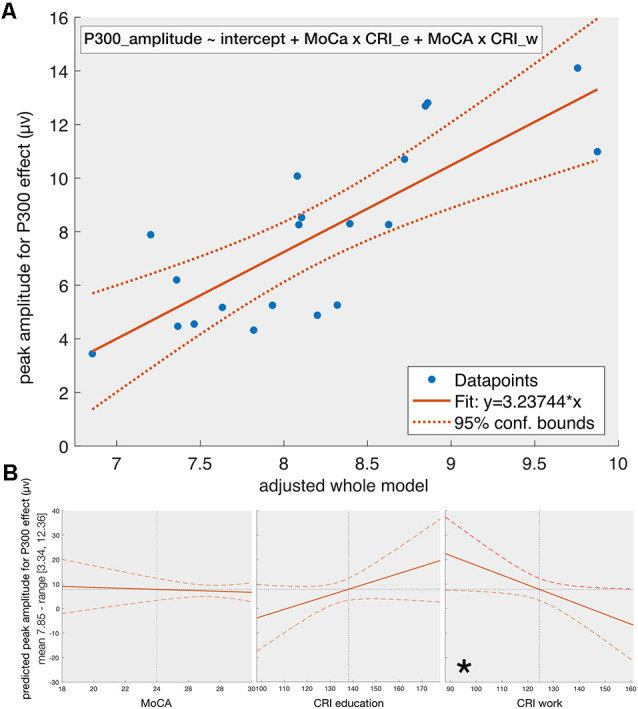
The fit of the whole model on P300-amplitude (Panel **A**) and the effect of individual predictors (Panel **B**) given that the other predictors are constant. Panel **(A)** shows the dependency of the amplitude of the P300-effect on the adjusted whole model (see equation 1) obtained from stepwise regression with MoCA, CRI_e, CRI_w, and CRI_l, and their first level interactions as predictors. The final model contains the MoCA*CRI_e and MoCA*CRI_w as predictors. The solid line represents the fit of the obtained model and the dotted lines reflect the 95% confidence intervals. Panel **(B)** reflects the effect of individual predictors included in the final obtained model on the peak amplitude of P300-effect given that the other predictors are constant. When all other predictors are kept constant, the only significant one is the CRI_w. This is because the predictors of the obtained model are interactions of the initial factors and the *p*-value for removal of predictors during the stepwise regression was set by default to 0.1 (see the section on statistics), which is higher than the *p-value* we set for statistical significance (0.05). CRI_e—the education score for CRI, CRI_w—the working attainment score for CRI, *indicating a significant relationship if the other predictors are kept constant.

For the peak amplitude (Figure 2), the stepwise regression results in the following model:

(1) P300_amplitude ∼ intercept+MoCA×CRI_e+MoCA×CRI_w

The adjusted-R^2^ is equal to 0.5 (*F* = 4.81, *p* = 0.009). The obtained model suggests a mostly non-linear relationship between P300 amplitude, MoCA and subcomponents of CRI: an ANOVA test on the obtained model shows a threshold significance for the linear model (*F*_(3,14)_ = 3.4, *p* = 0.047) and a strong significance for the non-linear model (*F*_(2,14)_ = 6.9, *p* = 0.0082). The component ANOVA test (considering all other predictors in the model constant except the one under consideration) on the obtained regression model shows a statistical significance for CRI-w subcomponent (*F*_(1,14)_ = 8.15, *p* = 0.013), as well as MoCA × CRI_e (*F*_(1,14)_ = 13.48, *p* = 0.0025) and MoCA × CRI_w (*F*_(1,14)_ = 11.89, *p* = 0.0039) interactions. Neither MoCA (*p* = 0.14), nor CRI_e (*p* = 0.67) alone shows statistical significance.

**Figure 3:**
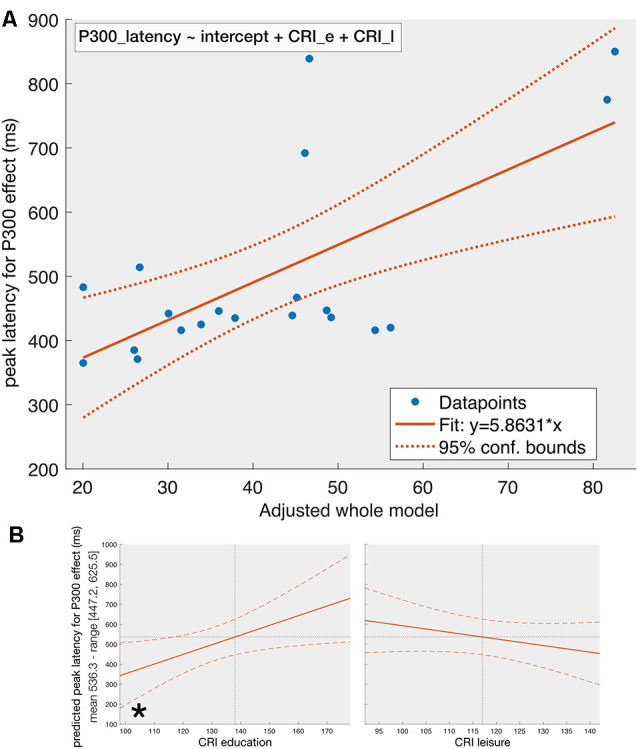
The fit of the whole model on P300-latency (Panel **A**) and the effect of individual predictors (Panel **B**) given that the other predictors are constant. Panel **(A)** represents the dependency of the peak latency of the P300-effect on the adjusted whole model [see equation (2)] obtained from a stepwise regression with MoCA, CRI_e, CRI_w, and CRI_l, and their first level interactions as predictors. The final model contains the CRI_e and CRI_l as predictors. The solid line represents the fit of the obtained model and the dotted lines reflect the 95% confidence intervals. Panel **(B)** reflects the effect of individual predictors included in the model on peak latency of P300-effect given that the other predictors are kept constant. When other predictors are kept constant, CRI_e is the only significant predictor in the model, which can be explained by noting that the *p*-value for removal of predictors during stepwise regression was set by default to 0.1 (see the section on statistics), which is higher than the *p-value* we set for statistical significance (0.05). Same conventions as in Figure 2.

For the peak latency of P300-effect (Figure 3), the stepwise regression with the same predictors reveals the following dependency:

(2) P300_latency∼intercept+CRI_e+CRI_l

The adjusted-R^2^ is equal to 0.38 (*F* = 6.82, *p* = 0.0067). Equation 2 suggests linear relationships between the peak latency of the P300-effect and CRI-subcomponents for education and leisure activities. The conducted ANOVA on the obtained model shows statistical significance for CRI_e (*F*_(1, 17)_ = 12.3, *p* = 0.0027) but not for CRI_l (*F*_(1, 17)_ = 3.73, *p* = 0.07) subcomponents when considering the other predictor in the model constant.

## Discussion

Obtaining an unbiased estimate of an individual’s CR level can help us to integrate an extra proxy in the workout of patients with memory complaints that would make their assessment more objective. In the current study, we showed that certain EEG characteristics can provide an objective CR measure. While this is in line with previous suggestions, unlike other studies (Speer and Soldan, 2015; Šneidere et al., 2020), the advantage of our approach is that we adopted an EEG task that employs minimal memory resources and therefore does not rely on the use of active working memory as this can introduce confounds. Furthermore, the assessment of CR was done using a questionnaire that encompasses the individual’s life experience rather than depend on a single proxy of CR such as working memory or fluid intelligence. Finally, by using different methods for EEG and CR assessments, we ensured the absence of indirect correlations where both EEG and CR-assessment would simply represent two aspects of the same (working memory) task and therefore would correlate with each other.

Even though several studies have reported relationships between the P300 component and CR (Šneidere et al., 2020), the involvement of the CR subcomponents in these relationships was not always unambiguous (Pavarini et al., 2018). For instance, while Amin et al. (2015) observed a positive correlation between the P300 amplitude (and a negative correlation between the P300-latency) and a memory recall task that was supposed to reflect CR, several other studies (de Miranda et al., 2012; Raggi et al., 2013; Alperin et al., 2014) did not observe a significant relationship between the P300 characteristics in response to an oddball paradigm and the individual’s demographic (e.g., education) or cognitive variables.

The current study suggests that both the peak amplitude and -latency of the P300 effect are proxies of CRI with amplitude non-linearly modulated by education level and working attainment, and latency linearly dependent on education level and leisure activities. This indicates an elaborate relationship with P300 and CR as a combination of several CRI-subcomponents was needed to reflect the P300 features. Positive relationships between the P300 amplitude and cognitive activity, albeit linear (Amin et al., 2015) or dichotomic (Hasan et al., 2016; Gutiérrez-Zamora Velasco et al., 2021), were previously reported. Such examples are the work of Hasan et al. (2016) where they observed a larger amplitude of P300 in response to a color recognition task in young adults with higher education levels compared to the ones with lower education. Furthermore, Gutiérrez-Zamora Velasco et al. (2021) observed a larger P300 amplitude in response to working memory (a modified Sternberg) task in young adults with higher CR for both low and high load conditions compared to the ones with lower CR.

Our latency results suggest a dependency on the individual’s education level and leisure activities throughout a lifetime. The analysis of the model subcomponents suggests a positive relationship between P300 latency and educational level (given a constant leisure activity), which is less conventional and seems to go against previous results where a higher cognitive activity implied shorter P300-latency (Amin et al., 2015; Miranda et al., 2020), albeit not all reported observations reached significance (de Miranda et al., 2012; Begum et al., 2014; Hasan et al., 2016). A possible explanation for this can be that our older participants were in a compensatory stage and were calling upon their CR to perform the task at a high level. This means that even if the longer P300-latency assumes a longer processing time, it would be a sign of a compensatory mechanism enabling efficient processing of information. This goes along with some previous studies on CR that suggest that individuals in the compensation stage would sacrifice the speed of performance for an increased accuracy in cognitive task performance (Christensen et al., 1997; Hultsch et al., 1999). Another potential explanation for the observed P300-latencies can be attributed to the adopted analysis technique (i.e., the cluster-based permutation). As this is a data-driven approach that considers the “best” data from the individual subjects and works around the problem of multiple comparisons, it does not reflect the spatial difference between the subjects since the algorithm identifies clusters based on significant differences in neural responses to target and standard stimuli independently of their spatial distribution. Note that this is particularly important for the P300 component as it is expected to experience a frontal shift with age (Soltani and Knight, 2000).

As the only consistently reported relationship between demographic variables and P300 latency is a positive correlation between P300 latency and age, we fitted a linear regression model on P300 latency with participant age as predicting factor (Supplementary Figure 4) and observed, as one could expect (Pavarini et al., 2018), a positive regression (adjusted *R*^2^ = 0.477, *F*_(2, 18)_ = 18.3, *p* = 0.00045).

The main limitation of the current study is the relatively small number of tested subjects. However, the size of our population is not much smaller compared to some other studies that evaluated CR with electrophysiological methods such as EEG and magnetoencephalography (López et al., 2014; Speer and Soldan, 2015; Moussard et al., 2016; Martínez et al., 2018) Furthermore, the main goal of our study was to show that the P300 amplitude and latency relate to the individual’s CRI subscores, obtained with an easy to use questionnaire, and does not have to be mediated *via* an explicit working memory task. In future studies, our models can be further developed using data from larger cohorts preferably with an additional focus on age and gender.

The current study also has clinical implications as the risk of AD-development for individuals experiencing subjective complaints on memory impairment (subjective cognitive decline—SCD) depends, among others, on their CR level (Yue et al., 2021). In these individuals, the neuropsychological tests typically show results that are still within the norm, rendering them difficult to diagnose. The presented relationships of EEG measurements with the CR level is a promising avenue but still requires further studies to validate it for the assessment of the risk of AD-conversion in individuals with SCD.

## Conclusion

The current study probed the relationships between the characteristics of the P300 event-related potential and the subcomponents of the CR index questionnaire (CRIq). We showed that even when using a non-memory task, the peak amplitude and latency of the P300 effect reflect the individual’s CR. While the relationship between the P300 latency and CRI-subcomponents on educational level and leisure activities is linear and additive, the association between the P300 amplitude and CRI-subcomponents (educational level and working attainment) is non-linear.

To the best of our knowledge, this is the first study that tackles the relationships between electrophysiological activity of the brain and an individual’s leisure activities and does so by using an unbiased data-driven approach.

## Data Availability Statement

The raw data supporting the conclusions of this article will be made available by the authors, without undue reservation.

## Ethics Statement

The studies involving human participants were reviewed and approved by Ethics Committee of the Leuven University Hospitals. The patients/participants provided their written informed consent to participate in this study.

## Author Contributions

EK designed the experiment. BW implemented the experiment. EK and BW collected the data. MP conducted the first round of the data analysis. BS and JT recruited the participants. EK wrote the first draft of the manuscript. MVH obtained the funding for the study. All co-authors equally participated in creating the final draft of the manuscript. All authors contributed to the article and approved the submitted version.

## Conflict of Interest

The authors declare that the research was conducted in the absence of any commercial or financial relationships that could be construed as a potential conflict of interest.
