# Supplementary material for: Electrophysiological Proxy of Cognitive Reserve Index
Source: Front Hum Neurosci. 2021 Jul 8;15:690856. doi: 10.3389/fnhum.2021.690856 (PMC8295460; doi:10.3389/fnhum.2021.690856)
Supplement: Supplementary file 1 [file Table_1.DOCX]

Title: Electrophysiological proxy of cognitive reserve index

Authors: Elvira Khachatryan, Benjamin Wittevrongel, Matej Perovnik, Jos Tournoy, Birgitte Schoenmakers, Marc M. Van Hulle


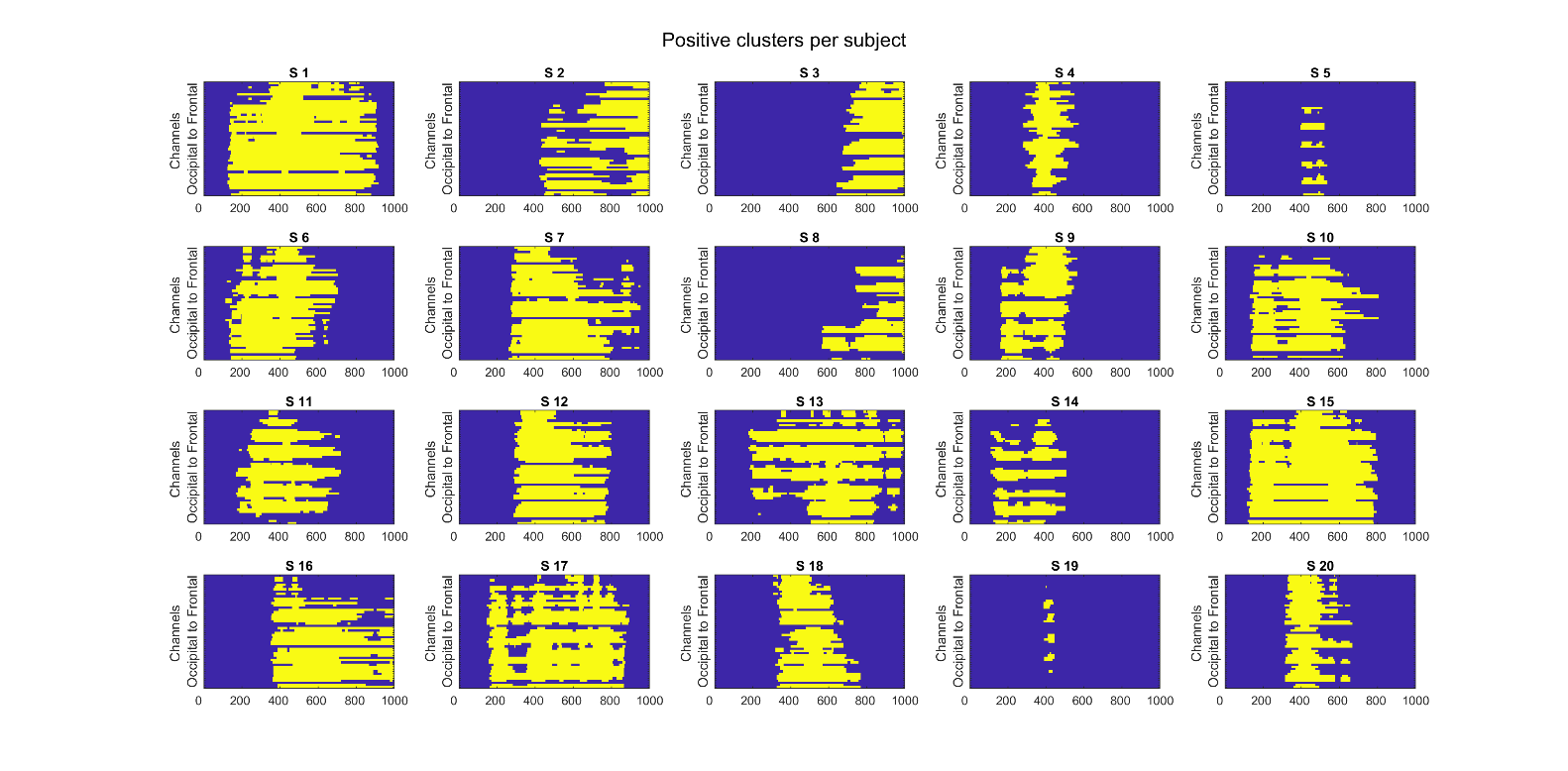


Figure S1: The largest positive cluster and its spatio-temporal distribution shown per subject


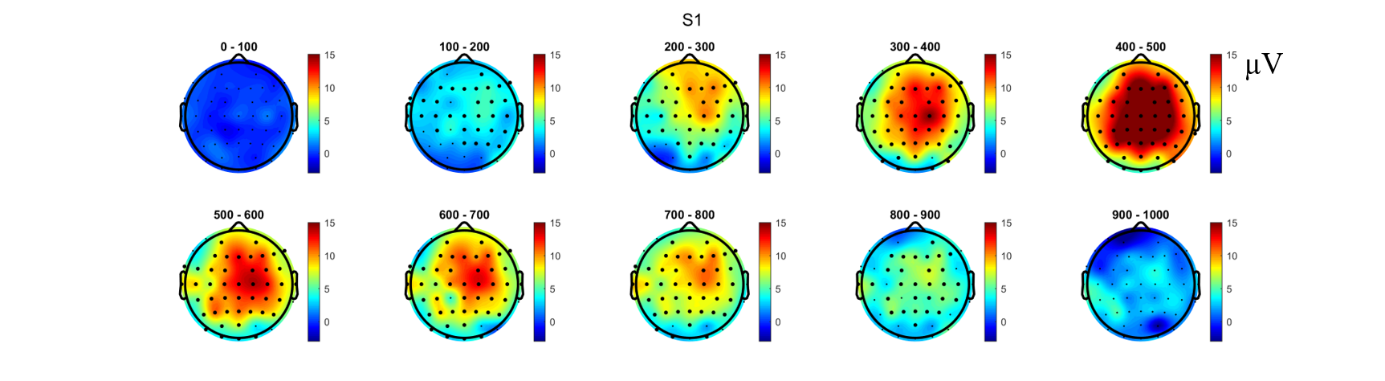


Figure S2: Temporal and spatial distribution of P300-effect in the largest cluster of subject S1. The per-electrode subtraction of averaged standard trials from the averaged target trials presented for S1 was done for illustrative purposes only. The electrodes that are included in the largest cluster are shown by thick dots.


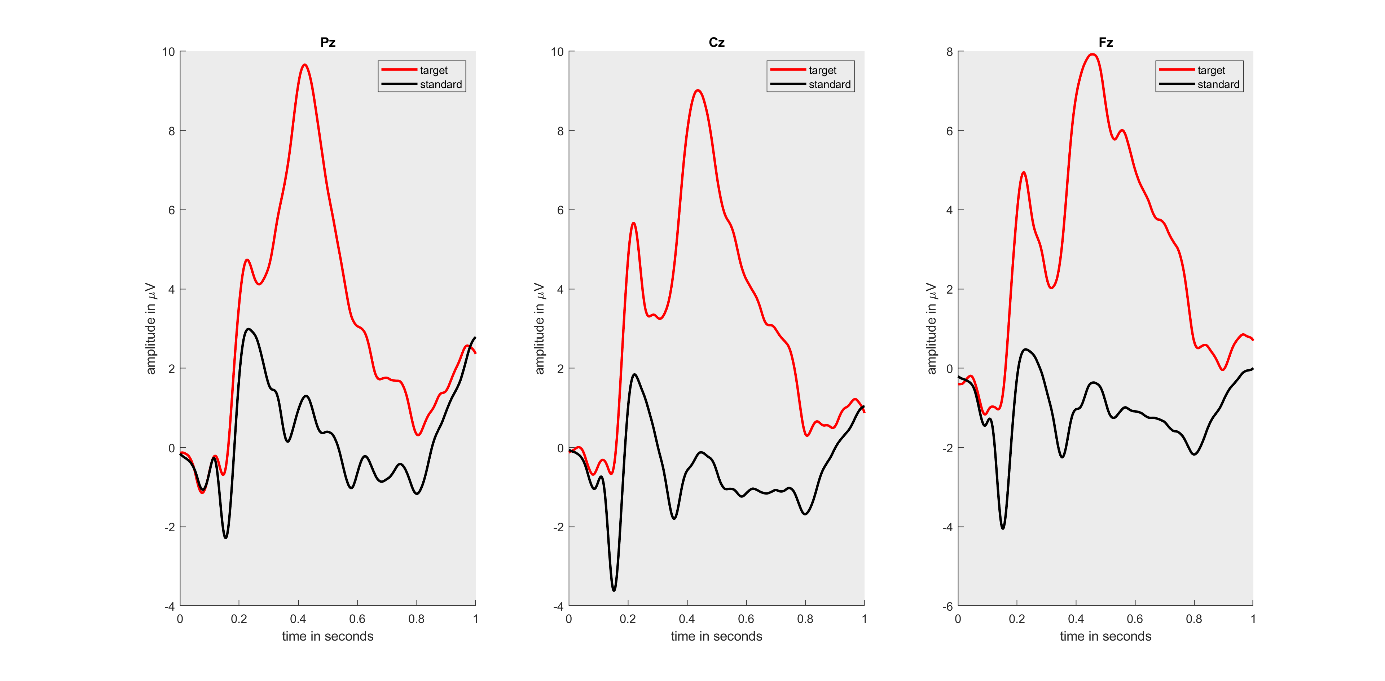


Figure S3: The ERP trace for the average of all subjects for electrodes Pz, Cz and Fz. Note that the averaging across subjects has been done for illustrative purposes only.

Figure S4: Scatterplot representing the dependency of P300 latency on participants’ age.
